# Improving HIV test uptake and case finding with assisted partner notification services

**DOI:** 10.1097/QAD.0000000000001555

**Published:** 2017-07-27

**Authors:** Shona Dalal, Cheryl Johnson, Virginia Fonner, Caitlin E. Kennedy, Nandi Siegfried, Carmen Figueroa, Rachel Baggaley

**Affiliations:** a Department of HIV/AIDS, World Health Organization, Geneva, Switzerland; b Department of Psychiatry and Behavioral Sciences, Medical University of South Carolina, Charleston, South Carolina; c Department of International Health, Johns Hopkins University Bloomberg School of Public Health, Baltimore, Maryland, USA; d Independent Clinical Epidemiologist, Cape Town, South Africa.

**Keywords:** contact tracing, couples, HIV, notification, partner

## Abstract

**Objective:**

Despite the enormous expansion of HIV testing services (HTS), an estimated 40% of people with HIV infection remain undiagnosed. To enhance the efficiency of HTS, new approaches are needed. The WHO conducted a systematic review on the effectiveness of assisted partner notification in improving HIV test uptake and diagnosis, and the occurrence of adverse events, to inform the development of normative guidelines.

**Methods:**

We systematically searched five electronic databases through June 2016. We also contacted experts in the field and study authors for additional information where needed. Eligible studies compared assisted HIV partner notification services to passive or no notification. Where multiple studies reported comparable outcomes, meta-analysis was conducted using a random-effects model to produce relative risks (RRs) or risk ratios and 95% confidence intervals (CIs).

**Results:**

Of 1742 citations identified, four randomized controlled trials and six observational studies totalling 5150 index patients from eight countries were included. Meta-analysis of three individually randomized trials showed that assisted partner notification services resulted in a 1.5-fold increase in HTS uptake among partners compared with passive referral (RR = 1.46; 95% CI: 1.22–1.75; *I*^2^ = 0%). The proportion of HIV-positive partners was 1.5 times higher with assisted partner notification than with passive referral (RR = 1.47; 95% CI: 1.12–1.92; *I*^2^ = 0%). Few instances of violence or harm occurred.

**Conclusion:**

Assisted partner notification improved partner testing and diagnosis of HIV-positive partners, with few reports of harm. WHO strongly recommends voluntary assisted HIV partner notification services to be offered as part of a comprehensive package of testing and care.

## Introduction

HIV testing and counselling services (HTS) and the availability of antiretroviral therapy have expanded enormously over the past three decades. Starting with diagnostic testing offered to people with symptoms suggestive of HIV infection and antenatal testing, HTS now encompasses a range of approaches such as community, home-based, and mobile testing to reach larger and more varied populations earlier in their course of infection. As a result, by the end of 2015, 17 million people with HIV infection were receiving antiretroviral treatment [1]. Yet it is currently estimated that over 14.5 million people living with HIV worldwide remain undiagnosed [2]. To address this gap – in particular, the first of the UN 90-90-90 goals to diagnose 90% of people with HIV infection by 2020 [2] – new approaches that enhance the efficiency of testing and increase the coverage of treatment are needed. HIV partner notification is an approach that has the potential to particularly identify people with undiagnosed HIV infection who remain unlinked to prevention, treatment and care services, and continue to be at risk of transmitting HIV vertically or through sexual and drug-injecting partners.

Assisted partner notification, or contact tracing has been an important public health approach in communicable disease management for decades, including in programmes for sexually transmitted infections (STIs) and tuberculosis (TB). The tracing of contacts and the voluntary screening of household members of patients with pulmonary TB is an effective and standard approach [3,4]. A 2013 Cochrane review found that expedited partner therapy was more successful than simple patient referral in preventing recurrent STIs causing urethritis or cervicitis [5]. Although it is well known that the sexual and drug-injecting partners of people diagnosed with HIV infection have an increased probability of also being HIV-positive [6–12], partner notification services for people diagnosed with HIV have not been routinely included in HTS policies internationally [13].

To inform a 2016 WHO HTS guidelines update, we conducted a systematic review and meta-analysis of partner notification services to determine their effectiveness in the uptake of HTS, diagnosing partners and linking them to care, and also to assess the occurrence of adverse events or harm following partner notification.

## Methods

We followed the methods described in the PRISMA statement for the reporting of systematic reviews and meta-analyses.

### Search strategy and inclusion criteria

Through 1 June 2016, we searched five electronic databases (PubMed, EMBASE, Cumulative Index to Nursing and Allied Health Literature CINAHL, PsycINFO, and Sociological Abstracts), and websites of major HIV-related conferences for relevant abstracts [International AIDS Conference (IAC), Conference on HIV Pathogenesis, Treatment, and Prevention (IAS), and Conference on Retroviruses and Opportunistic Infections (CROI)]. The IAC and IAS conference abstracts were searched for all available years; for CROI, only the most recent conferences (2014, 2015, and 2016) were searched as past conference abstracts were inaccessible online. In addition, selected experts in the field were contacted, and secondary reference searching was conducted on all included studies as well as on relevant review articles [5,14,15] to identify additional articles and abstracts. We also searched for ongoing randomized controlled trials (RCTs) through clinicaltrials.gov, the WHO International Clinical Trials Registry Platform (www.who.int/ictrp/), and the Pan African Clinical Trials Registry (www.pactr.org). We contacted study authors when additional information was needed.

A comprehensive PubMed search strategy was adapted for entry into all computer databases and included terms for HIV and partner notification and was not limited by study design: (HIV [tiab] OR ‘human immunodeficiency virus’ [tiab]) AND (‘contact examination’ [tiab] OR ‘contact detection’ [tiab] OR ‘contact tracing’ [tiab] OR ‘partner notification’ [tiab] OR ‘partner notifications’ [tiab] OR ‘partner tracing’ [tiab] OR ‘partner services’ OR ‘partner counseling and referral services’ [tiab]). No language or geographic limitations were placed on the search.

To be included, an article had to meet the following criteria: a study design that compared persons who received HTS and were diagnosed HIV-positive and who were offered partner notification services using assistance (such as contract or provider referral) to such persons who received HTS with passive referral or no partner notification intervention; measured one or more of the primary or secondary outcomes; and was published in a peer-reviewed journal or conference abstract.

Partner notification approaches included: first, passive referral, where HIV-positive clients are encouraged to disclose their status and suggest HIV testing to their partner(s) on their own; and assisted approaches: second, contract referral, where HIV-positive clients enter into a contract with a provider to refer their partner(s) to HTS within an agreed time period, after that the provider contacts the partner(s) directly and offers HTS, while maintaining the anonymity of the index patient. Third Provider referral, where providers directly contact partners of index patients to offer HTS, and fourth, dual referral where the provider accompanies the index patient when they disclose their status and offers HTS to their partner(s).

### Outcomes

Outcomes were: uptake of HTS among partner(s) of HIV-positive index patients; proportion of partners who tested for HIV and were diagnosed HIV-positive; any experience of social harm/adverse events among HIV-positive patients and/or their partners; measurement of CD4^+^ cell count or viral load among partners; linkage to clinical assessment or ART among partners following HIV-positive diagnosis; and linkage to a prevention visit among partners after an HIV-negative test result.

### Screening and data extraction

Screening was conducted in a two-stage process. First, titles, abstracts, and citation information identified through the search strategy were screened to remove clearly nonrelevant articles. Full-text articles for all selected abstracts were then screened by two independent reviewers for eligibility. Differences were resolved through consensus. Data were extracted into standardized coding forms.

### Statistical analysis

Data were analysed according to partner notification approach and outcome. Where multiple RCTs reported the same or comparable outcomes and were considered methodologically and clinically appropriate to combine, meta-analysis was conducted using a random-effects model to produce relative risks (RRs) (or rate ratios where applicable) and 95% confidence intervals (CIs) for dichotomous data using REVMAN version 5.3 (The Nordic Cochrane Centre, The Cochrane Collaboration, Copenhagen, Denmark). We conducted analyses using either all identified partners or all locatable partners as the denominator. For uptake of partner testing and linkage to care, we also analysed the rate ratio of partners tested to index patients to address the attrition of partners between those identified by index patients to those located and notified.

### Quality and Grading of Recommendations, Assessment, Development and Evaluation assessments

For individual RCTs, the risk of bias was evaluated using the Cochrane Collaboration's tool for assessing risk of bias [16]. We used Grading of Recommendations, Assessment, Development and Evaluation (GRADE) to determine the overall quality of evidence for each outcome measured in the RCTs. GRADE includes an appraisal of the risk of bias, imprecision, indirectness, inconsistency, and publication bias across included trials to inform an overall grading of high, moderate, low, or very low quality of evidence [17].

### Role of the funding source

The funders had no role in the development of this study. The authors alone were responsible for the study design, data collection, analysis, interpretation, and writing of the article. The corresponding author had the final responsibility for the decision to submit for publication.

## Results

**Fig. 1:**
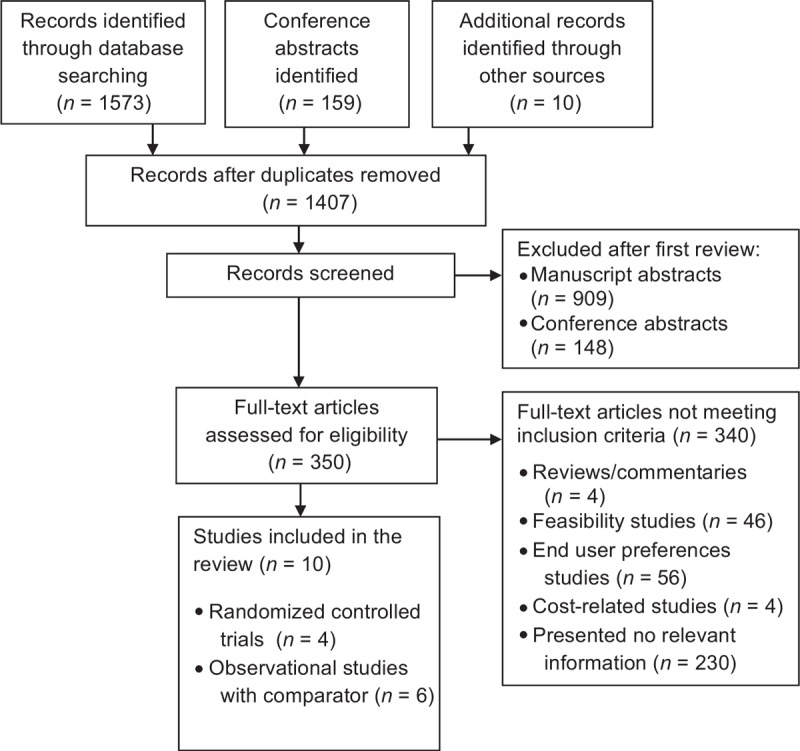
Study selection.

The searches yielded 1742 citations; four RCTs (three individually randomized trials and one cluster-randomized) met our eligibility criteria (Fig. 1). We included observational studies that compared types of partner notification services but either did not randomize index patients or did not have a nonintervention control arm, in order to provide an indication of broader geographic and population types for the main outcomes; these were not included in meta-analyses. For one cluster RCT [11] and observational study [18], we included results from a conference abstract in addition to results subsequently published in a peer-reviewed article [19,20], respectively, that were made available after the cut-off date for our initial search.

RCTs were conducted in the United States [8], Malawi [7,9], and Kenya [19]; the largest and most recent were in sub-Saharan Africa. Three RCTs compared assisted partner notification services (provider or contract referral) with passive approaches, and the fourth cluster RCT compared immediate assisted notification with a passive referral group that received delayed assisted partner notification after outcomes were assessed. The study populations included pregnant women attending antenatal care [9], patients from STI clinics [7], clients from an HIV testing centre [19], and patients in a United States county health department that included women, men who have sex with men (MSM), and people who inject drugs [8]. Six observational studies were conducted among the general population in Cameroon [21], Mozambique [20], Spain [22], the United Republic of Tanzania [23], and the United States [24], across a variety of healthcare settings and HIV testing sites (Table 1). All studies utilized multiple methods to contact and notify partners, including telephone calls, messages, and in-person visits.

**Fig. 2:**
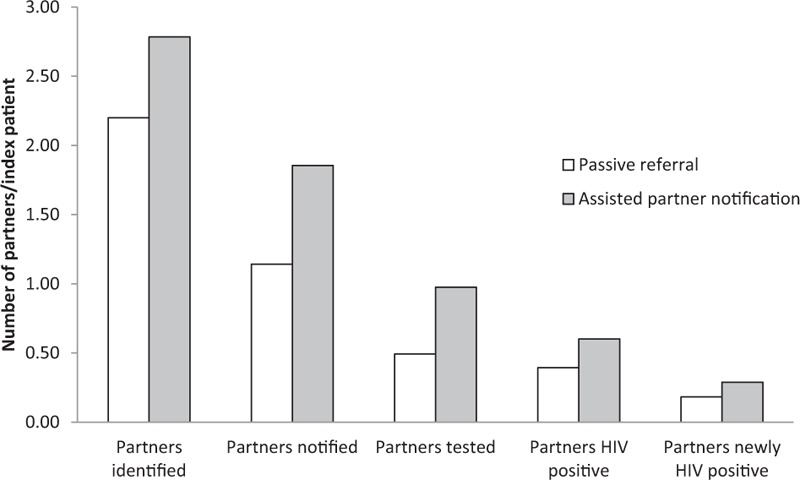
Partner notification cascade from five studies reporting data for each step [8,11,22–24].

The 10 studies included in our review were published between 1992 and 2016, and included a total of 5150 index patients who identified a total of 6127 partners (one study [9] provided only one partner invitation per index patient). On average, HIV-positive index patients named 2.0 partners, but this varied dramatically between studies (range 0.58–5.58); the largest were among key population groups (defined as MSM, people who inject drugs, sex workers, people in prisons, or transgender people). In the nine studies reporting outcomes by approach, the ratio of partners who tested for HIV per index patient was on average 0.45 (range 0.01–1.19) for passive referral, and 0.85 (range 0.19–1.81) for assisted partner notification (Table 1). A partner notification cascade using five studies which reported each step starting from the ratio of partners identified through to the number of partners newly identified as HIV positive per index patient, shows the progressive loss to follow-up of partners, the largest being between partner identification and notification (Fig. 2). On average, 1.14 (range 0.26–4.37) partners were notified per index patient following passive referral and 1.86 (range 0.93–4.03) with assisted notification.

**Fig. 3:**
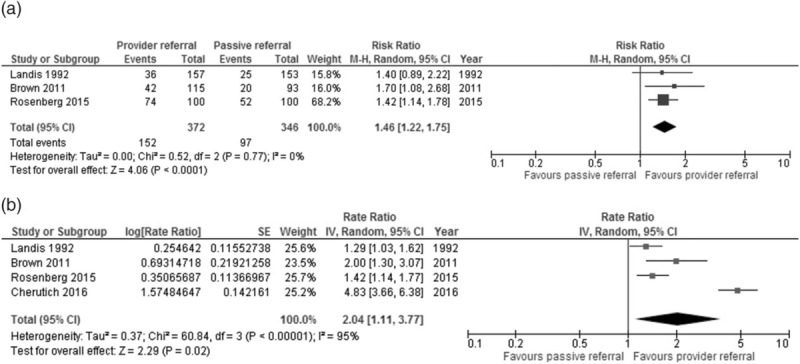
Uptake of HIV testing among partners of index cases assessed with: (a) HIV testing and return to clinic – meta-analysis using all identified partners as the denominator. (b) Rate of partner test or return to clinic of partner per index patient – meta-analysis using generic inverse variance.

Meta-analysis of the three individually randomized trials using all identified partners as the denominator, showed that assisted partner notification services resulted in a 1.5-fold increase in the uptake of HTS among partners compared with passive referral (RR = 1.46; 95% CI: 1.22–1.75; Fig. 3a) [7–9,19]. Meta-analysis restricted to partners who could be located in the denominator found a similar beneficial effect. Statistical heterogeneity was high (RR = 1.39; 95% CI: 0.93; 2.06; Chi^2^ for heterogeneity = 8.34; df = 2; *I*^2^ = 76%) (Appendix Fig. A1). When all four RCTs were included in a meta-analysis of the rate of partner testing and return of partners to the clinic *per index case*, the rate ratio of the assisted partner notification group was twice that of those in the passive referral group (rate ratio: 2.04; 95% CI: 1.11–3.77; chi^2^ for heterogeneity = 60.84; df = 3; *I*^2^ = 95%) (Fig. 3b). GRADE quality evidence was rated moderate for all analyses of HIV testing uptake due to the lack of blinding across studies, and attrition. In five observational studies, assisted partner notification was associated with increased uptake of HTS among identified partners compared with passive referral [20–22,24].

**Fig. 4:**
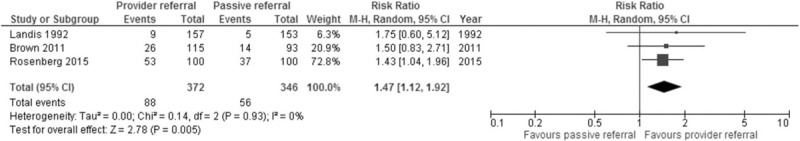
Proportion of partners who tested and were diagnosed HIV positive – meta-analysis using all identified partners as the denominator.

The proportion of partners of index patients who tested HIV-positive ranged from 20 to 72% in both passive and assisted arms of the four trials (Table 1) [7–9,19]. Among the observational studies, the highest proportion testing HIV positive was 86%. In the four studies [7,9,20,21] that reported on couples, between 29 and 40% were in serodiscordant partnerships. A meta-analysis of the three individually RCTs found that the proportion of all identified partners who were HIV-positive following testing was 1.5 times higher in the assisted partner notification approach than in the passive approach (RR = 1.47; 95% CI: 1.12–1.92) (Fig. 4). In sensitivity analyses, the results were similar using locatable partners as the denominator (RR = 1.49; 95% CI: 1.14–1.95) (Appendix Fig. A2).

The percentage of partners *newly* diagnosed with HIV among partners who could be located, was higher with provider assisted partner notification (RR = 1.37; 95% CI: 0.98–1.93) (Appendix Fig. A6), and was similar when the cluster RCT was included in analyses with high statistical heterogeneity (Appendix Fig. A3). Across the observational studies, 0 to 86% of partners of HIV-positive individuals were newly diagnosed with HIV [20–24]. An observational study in Mozambique reported a two-fold increase in the percentage of partners diagnosed with HIV when passive approaches were replaced by assisted partner referral [20].

Meta-analysis of the two trials which reported on linkage to care showed that there was a higher rate of linkage to care in HIV-positive partners in the provider referral arm than in the passive arm (rate ratio = 3.76; 95% CI: 2.41–5.86; chi^2^ for heterogeneity = 1.48; df = 1; *I*^2^ = 33%)) (Appendix Fig. A4) [9,19].

All four trials and two observational studies reported few (0–3%) instances of harm resulting from partner notification [7–9,19–21]. A meta-analysis of two individually randomized and one cluster-randomized trial, showed no difference in social harm or adverse events comparing assisted and passive partner notification (RR = 1.86; 95% CI: 0.37–9.50) (Appendix Fig. A5). Reported incidents of harm in RCTs in Kenya and Malawi appeared not to be associated with HIV partner notification services, as they occurred prior to the intervention [9,19].

A single RCT [7] compared contract referral with passive referral. The quality of evidence was graded as very low for all outcomes as it was a single study, there was a lack of blinding of staff and participants, and results were imprecise. Results showed that assisted partner notification services using contract referral resulted in a two-fold increase in test uptake among the partners of HIV-positive individuals compared with passive referral (RR = 2.08; 95% CI: 1.33–3.25). The proportion of identifiable partners who tested for HIV and were diagnosed HIV positive was higher for contract referral than passive referral (RR = 1.91; 95% CI: 1.07; 3.40). Sensitivity analyses were comparable (data not shown).

## Discussion

When HIV positive index patients were offered assistance in notifying their sexual and drug-injecting partners of their exposure to HIV infection, our analyses show that it resulted in higher uptake of partner HIV testing, identified higher proportions of HIV-infected persons, and increased linkage to care through the referral of newly identified HIV-infected partners to ART services. Although there were few RCTs in our meta-analyses, the results are consistent towards favouring assisted approaches, as are the results from observational studies with control groups. Across all studies, high proportions of partners returned for HIV testing when contacted by a provider, whichever method was used. Overall, index patients identified an average of two partners each and this resulted in 0.44 (range 0.01–1.8) partners per index patient eventually testing, following attrition between identified and notified partners, and acceptance of testing. The proportion of partners who tested HIV positive following assisted notification across all studies ranged from 12 to 86%, and between 29 and 40% of couples were serodiscordant.

Although all the studies that were reviewed showed improved outcomes with assisted partner notification approaches in both RCTs and observational studies, passive referral also resulted in uptake of HIV testing among partners (range 2–65%) [7–9,20,22,23]. In some studies, HIV test uptake in the passive group was seen at a similar or higher level to that of assisted approaches from other studies. Two studies with very low HTS uptake in the passive referral groups were conducted in the United States before triple therapy was available (3% testing uptake) [8], and when implementation of partner notification reporting regulations appeared to be low (2% test uptake) [24]. The remaining studies presenting this information reported HIV test uptake between 24 and 65% [7,9,19,20,22,23,25]. Furthermore, observational data from Cameroon demonstrate the scalability of partner notification with the offer of multiple notification approaches to index patients in a programmatic setting, resulting in high partner test uptake overall (67%) [21]. Thus, the simple act of encouraging partner notification and offering services to a person who is HIV positive, whether verbally during counselling, or through written invitation letters or referral cards, is beneficial, and could be considered while assisted approaches are being brought to scale. Linkage to care for partners who test HIV positive was higher with assisted partner notification methods than with passive methods in both RCTs [9,19] and observational studies [21,23].

The ratio of partners tested per index patient varies, but in all studies, a substantial drop occurs between identified and notified partners and may be due to the difficulty in contacting partners. One of the challenges to partner notification has been that key populations [26] and people with casual partners [7,27] may be less able or willing to identify partners; spouses and steady partners have been more likely to be notified than other partners [7,21,28–30]. Recall of, and contact information for, partners was reported to be better among heterosexual women than among MSM or people who inject drugs in one study [31]. Yet, as was found in studies conducted among the general population, assisted partner notification services among key populations resulted in higher uptake of HTS, and particularly among MSM and people who inject drugs, also identified a high proportion of HIV-positive partners (5–80%) [32–54]. A recently published observational study found that 36% of newly diagnosed partners had acute or early HIV infection, and among partners with genetic sequences, 61% were genetically linked to the index patient, emphasizing the importance of reaching partners to prevent transmission in discordant partnerships [55]. Providing partner notification to key populations and those with casual partners may require more intensive efforts to locate partners, including the assurance of confidentiality and anonymity for HIV-positive clients.

Reported social harm and other adverse events following HIV partner notification, using passive or assisted approaches, have been rare. Fears about social harm following disclosure or partner notification are of particular concern in situations where certain behaviours associated with HIV infection are criminalized, such as among people who inject drugs, or where one partner is economically dependent on the other and fears losing social or financial support. However, although issues around confidentiality [56], and mostly hypothetical concerns about potential harm [57,58] have been raised in the literature, when adverse events have actually been measured, very few have occurred [7–9,19]. Moreover, studies from the United States showed no differences in partnership dissolution following HIV partner notification when compared with a high-risk HIV-negative control group [59], or to syphilis partner notification [28]. Some studies screened for intimate partner violence (IPV) and excluded those persons with a history of IPV, which would put them at risk of harm following disclosure. Reported incidents of harm in RCTs in Kenya and Malawi appeared not to be associated with HIV partner notification services, as they occurred prior to the intervention [9,19]. Although programme implementers should be sensitive to the potential for harm arising from disclosure of HIV status and assisted partner notification, this should be balanced against the benefit of diagnosing HIV infection and linking people to treatment. These results were obtained from a limited number of studies undertaken in the United States and Africa; studies from other world regions are needed.

Our review identified four RCTs with which to assess the effectiveness of HIV partner notification services with the quality of evidence for the primary outcome graded as moderate. For some outcomes, significant statistical heterogeneity was present, mostly driven by the large effects observed in the cluster-randomized trial. We conducted sensitivity analyses to test the robustness of the results, using different denominators and methods of outcome measurement and found consistent results. One trial [8] was conducted before the advent of combination antiretroviral therapy and was the only one which included key populations, and assessed outcomes by two assisted approaches (provider and contract referral); excluding it would have strengthened the impact of partner notification interventions. Although included data were derived from RCTs, evaluation of the quality of the evidence using the GRADE approach identified a high risk of performance and detection bias due to a lack of blinding across trials, attrition, and in some instances, imprecision and data arising from a single study. Despite these limitations, our result for the main outcome of HIV test uptake was clear and consistent, with observational data also indicating that assisted partner notification was beneficial. The pooled synthesis on social harm indicated very few events. The quality of evidence was downgraded due to imprecision and risk of bias, but when considered with similarly few adverse events from observational data, it suggests that the rate of social harm is not likely to differ between assisted and passive partner notification approaches.

In conclusion, our findings show that assisted partner notification increased HIV test uptake and diagnosed high proportions of people with HIV infection, with very few reports of harm. The difficulty in tracing identified partners may have resulted in the low ratio of partners notified per index patient. However, the high proportion of partners who were HIV positive among those who were notified warrants the efforts needed to reach partners for testing. Furthermore, treatment for infected partners is critical to prevent transmission to seronegative partners for those in serodiscordant partnerships. Assisted HIV partner notification should be implemented as a routine part of HTS and should be offered to all newly diagnosed persons, and periodically to all HIV positive persons throughout their care and treatment.

## Acknowledgements

We thank Ping Teresa Yeh, Caitlin Payne, and Sophie Morse for their assistance with the literature search and screening process, and the WHO guideline development group members: Kindi Adam, Oliver Anene, Karen Champenois, Kathleen Charters, Martin Choo, Miriam Franchini, Rebecca Guy, Mehdi Karkouri, Dasha Matyushina Ocheret, Getrude Ncube, Sabin Nsanzimana, Bathabile Nyathi, Carla Obermeyer, Niluka Perera, Archana Sarkar, Jennifer Stuart-Dixon, Joseph Tak Fai Lau, Jane Thiomi, Francois Venter, and Vincent Wong. Thanks also to the WHO guideline steering committee members: Wale Ajose, Annabel Baddaley, Michel Beusenberg, Brian Chirombo, Lastone Chitembo, Rosalind Coleman, Meg Doherty, Philippa Easterbrook, Shaffiq Essajee, Haileyesus Getahun, Peter Godfrey-Faussett, Joumana Hermez, Naoko Ishikawa, Lali Khotenashvili, Daniel Low Beer, Frank Lule, Christine Mushanu, Simbarsha Mabaya, Buhle Ncube, Augustine Ntilivamunda, Ishmael Nyasulu, Martina Penazzato, Carmen Perez Casas, Julie Samuelson, Anita Sands, Willy Urassa, Freddy Perez, Razia Pendse, Bharat Rewari, Ying Ru Lo, Mukta Sharma, Nicole Seguy, Annette Verster, and Teodora Wi.

The current systematic review was supported with funding by the Bill and Melinda Gates Foundation and the United States Agency for International Development.

Contributors: R.B. and C.J. conceptualized the study. C.E.K. and V.F. conducted record screening and led data extraction. S.D., N.S., and C.J. extracted additional data. S.D. and N.S. conducted statistical analyses. S.D. drafted the article. C.F. and R.B. provided methodological and contextual input. All authors reviewed and approved the final article.

### Conflicts of interest

There are no conflicts of interest.

## Supplementary Material

Supplemental Digital Content

## Figures and Tables

**Table 1:** Study descriptions and HIV partner notification outcomes for studies included in this review.

					Passive/control groups^a^					Assisted groups (includes provider, contract, and dual referral)				
Author, year	Country	Study design	Population	Intervention	Number index cases	Number partners identified	Partners tested (%)	Partners HIV positive (%)	Ratio of partners tested to index case	Number index cases	Number partners identified	Partners tested (%)	Partners HIV positive (%)	Ratio of partners tested to index case
Landis *et al.*, 1992	USA	RCT	County health dept women, MSM, PWID	Passive referral vs. mix of provider and contract referral	35	153	5 (3)	1 (20)	0.14	39	157	36 (23)	9 (25)	0.92
Brown *et al.*, 2011	Malawi	RCT	STI clinic patients	Passive, vs. contract vs. provider referral	77	82	20 (24)	12 (60)	0.26	163	170	87 (51)	42 (48)	0.53
Rosenberg *et al.*, 2015^b^	Malawi	RCT	Pregnant women	Passive referral vs. contract referral	100	NA	52 (52)	37 (71)	0.52	100	NA	74 (74)	53 (72)	0.74
Cherutich *et al.*, 2016	Kenya	Cluster-RCT	HIV testing centre clients	Immediate vs. delayed PN^c^	569	959	85 (9)	28 (33)	0.15	550	913	392 (43)	136 (35)	0.71
Udeagu *et al.*, 2012	USA	Preintervention–postintervention	STI clinic patients	Preinitiative (2005) vs. post-PN program (2008)	670	174	4 (2)	0	0.01	602	562	117 (21)	15 (13)	0.19
Plotkin *et al.*, 2015	United Republic of Tanzania	Cross-sectional	3 hospitals	Offer of passive, provider, or contract referral	356	402	241 (60)	142 (59)	0.68	14	16	7 (44)	6 (86)	0.50
Chiou *et al.*, 2015	Taiwan	Other^d^	MSM	1 session PN counselling vs. 2 sessions	42	165	33 (20)	9 (27)	0.79	43	302	78 (26)	31 (40)	1.81
Valle *et al.*, 2015	Spain	Observational	Healthcare settings	Offer of passive or provider referral	84	153	100 (65)	21 (21)	1.19	24	46	41 (89)	5 (12)	1.71
Henley *et al.*, 2015^e^	Cameroon	Observational	ANC, VCT and inpatients	Offer of passive, provider, and contract referral	592	423	191 (45)	–	0.32	870	1184	709 (60)	–	0.82
Myers *et al.*, 2016	Mozambique	Observational	Clinic patients	4 weeks passive referral followed by 4 weeks contract referral in same index patients^f^	206	262	82 (31)	34 (41)	0.40	NA	NA	83 (32)	43 (52)	–

ANC, antenatal clinic; Dept, department; NA, not applicable; PN, partner notification; PWID, people who inject drugs; RCT, randomized controlled trial; STI, sexually transmitted infection; VCT, voluntary counselling and testing.

^a^We present the data for delayed PN, diagnosis in 2005, patient choice of passive referral, passive phase, and one PN counselling session in the unassisted columns.

^b^Study provided one partner invitation card per index client, so the maximum number of partners possible was 100.

^c^Passive referral group received delayed assisted partner notification after outcomes were assessed.

^d^Study used random assignment of participants to either one or two partner notification counselling sessions, and participants in both arms were offered passive, provider, contract, or dual referral for notifying partners. Therefore, it did not meet trial inclusion criteria.

^e^Total sample size of index clients = 1462, but data were not available by arm. Data from 107 participants who did not choose a PN type, but where partners were notified and tested, were included in the passive referral group.

^f^Index patients first received passive referral for partners for 4 weeks, followed by contract referral for an additional 4 weeks for partners who had not been notified in phase I.
