# Supplementary material for: Improving HIV test uptake and case finding with assisted partner notification services
Source: AIDS. 2017 Jul 27;31(13):1867–76. doi: 10.1097/QAD.0000000000001555 (PMC5538304; doi:10.1097/QAD.0000000000001555)
Supplement: Supplemental Digital Content [file aids-31-1867-s001.docx]

**Supplementary Appendix. Sensitivity analyses of HIV test uptake and proportions diagnosed HIV positive among partners of index cases comparing provider referral to passive referral**

Figure A1 Uptake of HIV testing among partners assessed with HIV testing and return to clinic – Meta-analysis using locatable partners as denominators

**
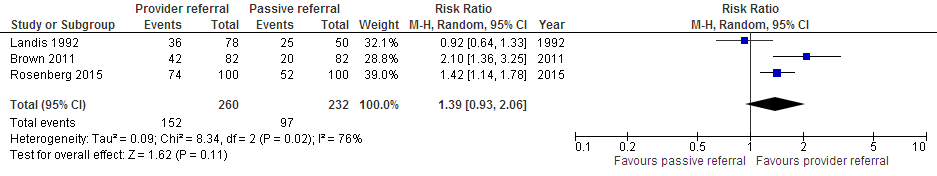
**

Figure A2. Proportion of partners who tested and were diagnosed HIV positive – Meta-analysis using all locatable partners as the denominator

**
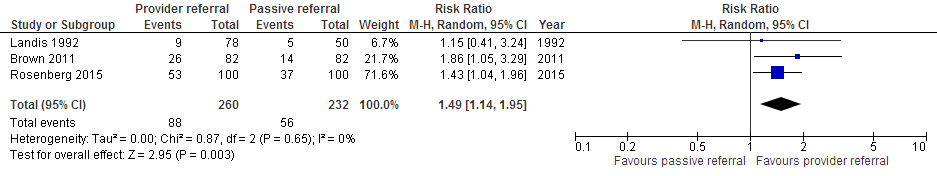
**

**Figure A3. Proportion of partners who were *newly* diagnosed HIV positive – Meta-analysis using generic inverse variance among locatable partners**

**
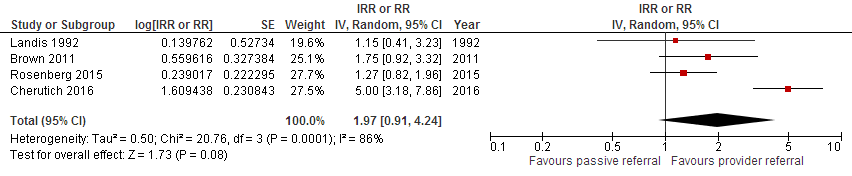
**

**Figure A4. New linkage to care among HIV positive partners – Meta-analysis using generic inverse variance**

**
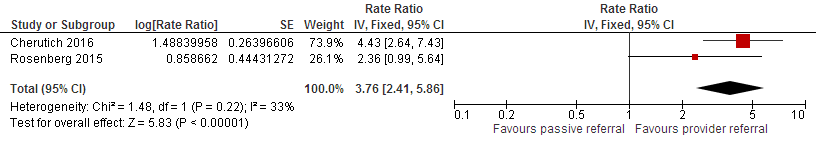
**

Figure A5. Adverse events assessed with intimate partner violence or abandonment using locatable partners as denominator

**
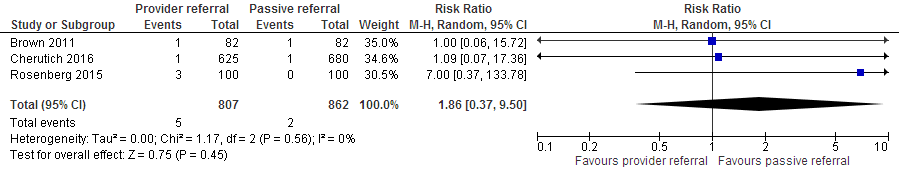
**
